# Rigor Mortis in Farmed Common Carp *Cyprinus carpio*: The Effects of Handling Temperature

**DOI:** 10.1155/ijfo/8376835

**Published:** 2025-04-29

**Authors:** A. A. Mohammed, A. Y. Jassim, A. T. Yesser, Q. H. Al-Hamadany

**Affiliations:** Marine Vertebrates Department-Marine Science Center-University of Basrah, Basrah, Iraq

**Keywords:** ambient temperature, chemical composition, chilled temperature, common carp, rigor mortis

## Abstract

The study was conducted to determine the rigor mortis in common carp *Cyprinus carpio*, weighing 1100 ± 163 g and measuring 37.5 ± 1.87 cm in total length. Immediately after catching, the fish were killed by a blow to the head, followed by washing with tap water, before being placed in six insulated boxes, either at the ambient temperature (25 ± 3°C) or at a chilled temperature of (0°C). The rigor mortis and biochemical changes were monitored at regular times to measure the rigor mortis index (%), pH, and chemical composition. The rate of rigor mortis index in fish kept at ambient temperature was 100% within 10 h after fishing, while it was 100% after 20 h in fish kept at a chilled temperature. During the later postmortem changes, pH slightly increased to reach a value of 7.2 for fish kept at ambient temperature and 7 for fish kept at a chilled temperature. The results also showed nonsignificant differences (*p* > 0.05) in the chemical composition of fish before and after rigor mortis. Therefore, it was considered that the development of rigor mortis in common carp occurring more rapidly at ambient temperature than at chilled temperature.

## 1. Introduction

Common carp fish is one of the most important farmed fish in Iraq because of its flavoring taste, valuable size, high market demand, rapid growth, disease resistance, and adaptability to a wide range of environmental conditions in all common culture systems, especially earthen ponds, and cages [1]. The quality of the muscles in any aquatic organism, including fish, crabs, and mollusks, has been found to decline immediately after capture and death [2]. Fish go through a variety of biochemical changes following death, such as rigor mortis, autolysis, the breakdown of adenosine triphosphate (ATP), a drop in pH, and protein denaturation [3]. Reducing postharvest losses and enhancing fish marketing require an understanding of the quality of fish species that are essential for economic use. The quality of fish is affected by several factors, with rigor mortis being the most prominent one, which develops after death [4]. Assessment of rigor mortis provides an appropriate approach to determine fish freshness during the Fish in early storage, whether prerigor or full rigor. are considered very fresh [5]. Many different factors can affect how rigor mortis develop. The duration of time it takes for a fish to enter and recover from rigor mortis depends on its species, size, physical condition, level of stress before death, handling, and the temperature at which it is stored [6]. There is a relationship between rigor mortis and keeping the quality of fish. The keeping quality of fish depends largely on the duration of the prerigor period [7]. Fish flesh maintains its freshness throughout the prerigor period. Degeneration starts during the postrigor phase and the early stages of relaxation. Typically, a slower rate of rigor mortis progress results in a longer shelf-life for the fish. Since fish in a prerigor state are valued equally to live fish in the market, delaying the onset of rigor mortis is very critical [8, 9]. It is commonly believed that reducing the temperature prevents rigor mortis from progressing and delays its onset [6]. It is advisable to prolong the rigor mortis stage to extend the shelf life of fish. It was found that in tropical or temperate regions, changes in the chemical composition of fish and the state of rigor mortis are better at 0°C compared to room temperature [10]. The status of rigor mortis in farmed common carp fish is examined for the first time in this study by comparing the chemical and biochemical changes of rigor mortis before and after rigor mortis. Multiple techniques for assessing rigor mortis have been reported. Direct measurements rely on the observation of alterations in physical and mechanical properties such as rigor index [11], shear strength [12], and isometric muscle strength [13, 14].

Common carp, *Cyprinus carpio*, are a major species that are farmed in Iraq. Studies on the postmortem changes to its muscle during handling and storage are still limited. The objective of the study is to identify postmortem changes in common carp fish muscle during postcatch to determine how freshness and decomposition indices have changed over time. The findings will be applied to define quality criteria for this species, and they should help fish farmers and processors create more successful processing and marketing strategies.

## 2. Materials and Methods

### 2.1. Fish Samples

Then, 60 live common carp fish with an average weight of (1100 ± 163 g), and average length (37.5 ± 1.87 cm), were obtained from the Marine Science Center education fish farm at the University of Basrah, Basrah–Iraq, transported to Laboratories of Marine Vertebrates Department, Marine Science Center, University of Basrah. The fish were immediately killed by a blow to the head, followed by washing with tap water, and then either placed in an insulated box with a fish-to-ice ratio of 1:2 or in an insulated box at ambient temperature (25 ± 3°C) until subsequent experiments.

### 2.2. Rigor Mortis Index Measurement

The rigor mortis index of the fish was assessed by observing and measuring the curvature of the tail, as described in the method developed by Bito [11]. To carry out this measurement, the fish was carefully positioned on a flat horizontal surface, such as a table. The setup required that the body of the fish, beyond the point of the posterior dorsal fin, be allowed to hang freely over the edge of the table. The rigor mortis index was expressed as a percentage and determined using a specific formula, which is as follows:

 $$\begin{array}{c} \text{Rigor mortis index }\left(\%\right)=\left[\frac{\;D_{0}-D}{D_{0}}\right]\times 100. \end{array}$$

In this formula, two key measurements, *D*_0_ and *D*, were used. *D*_0_ represents the initial distance measured from the horizontal surface of the table to the base of the tail before rigor mortis occurred, while *D* represents the corresponding distance after rigor mortis had set in.

### 2.3. pH Measurement

pH was measured at regular intervals of 0, 5, 10, 15, 20, 25, 30, 35, and 40 h with a pH meter (Lovibond-pH 200). Then, 10 g of fish meat and 30 mL of distilled water were blended in an electric blender for 1 min. The mixture was homogenized for 5 min before the reading was taken.

### 2.4. Chemical Composition

Following the procedures given by AOAC [15], the percentages of protein, fat, moisture, and ash in fish muscles were measured before and after the onset of rigor mortis.

### 2.5. Statistical Analysis

Data was analyzed using SPSS software (SPSS 26.0 for Windows, SPSS Inc.) to explore the statistical significance of the results. CRD (completely randomized design) was employed. The data were analyzed using analysis of variance (ANOVA). Subsequently, for all parameters, comparisons between means were performed with Duncan's multiple range test at a significant level of 0.05.

## 3. Results

### 3.1. Rigor Mortis Index

**Figure 1:**
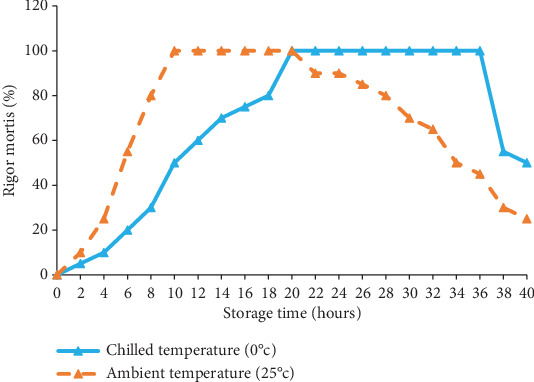
Rigor mortis (%) of common carp held at chilled temperature (0°C) and ambient temperature (25°C).

The rigor mortis index for common carp held in a chilled and ambient temperature is shown in Figure 1. The progress of rigor mortis at ambient temperature is exhibited faster compared to fish held in ice. For fish held at ambient temperature, rigor mortis started 2 h after death, and it reached a maximum of 100% within 10 h. The maximum rigor stage lasts about 10 h and then starts to postrigor. The fish fully relaxed after 34 h.

Conversely, in chilled fish, rigor mortis started within 4 h. However, the progress rate was much slower than that of the fish held at ambient temperature. Rigor mortis increased gradually over time. The rigor mortis progressed to a maximum of 100% after 20 h. They remained in this condition for 18 h and then began to relax from rigor. The rigor relaxed for up to more than 40 h for both treatments.

### 3.2. Changes in pH

**Figure 2:**
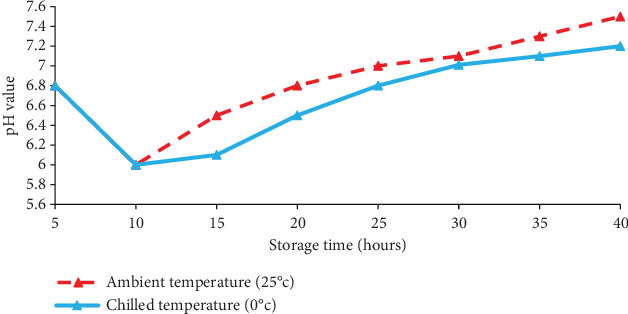
Changes in pH of common carp held at chilled temperature (0°C) and ambient temperature (25°C).

The muscle pH of common carp fish immediately after death was close to neutral (6.8). pH decreased to 6.0 during the early hours of rigor mortis (Figure 2). During the later postmortem changes, pH slightly increased to 7.2 and 7.0 in fish stored at ambient and chilled temperatures, respectively.

### 3.3. Changes in Chemical Composition

The result of the proximate composition of common carp fishes before and after rigor mortis was summarized in Table 1. No significant differences (*p* > 0.05) were found in the chemical composition of common carp before and after rigor mortis. On a wet weight basis, moisture, protein, lipid, and ash content were 76.98 ± 1.04%, 15.92 ± 0.84%, 5.42 ± 0.27%, and 1.86 ± 0.03%, in fish before rigor mortis, respectively, while it was 76.71 ± 1.23%, 15.41 ± 0.53%, 5.94 ± 0.31%, and 1.93 ± 0.06% for moisture, protein, fat, and ash, respectively, in fish kept at ambient temperature after rigor mortis, and 76.82 ± 0.97%, 15.23 ± 0.58%, 5.87 ± 0.23%, and 1.98 ± 0.07%, respectively, in chilled fish after rigor mortis.

## 4. Discussion

After being caught, fish undergo some internal changes that ultimately lead to spoiling, a drop in quality, and restrictions on fish consumption. These changes encompass rigor mortis, autolysis, and lipid oxidation, as well as bacterial and chemical changes. The postmortem physical conditions of the fish and the rate of temperature change it is subjected to can impact the time the fish enters or exits from a state of rigor mortis [16]. Theoretically, increased storage temperatures would accelerate the development of postmortem, biochemical changes, and rigor mortis [17]. Keeping fish in ice has been introduced as one of the essential methods for preserving and maintaining the freshness of fish.

Additionally, chilled storage lowers the rates of microbiological, chemical, and enzymatic activities [18]. Lower temperatures have been shown to delay the onset of rigor mortis and to slow the rate of its progression [8]. The results obtained from the current study agree with those reported for other fish under different storage conditions. According to [19], rigor mortis started to develop in fish kept at storage temperatures of 28°C and 30°C an hour after fish died, but it lasted for a very long time in fish kept in ice. Rigor index reached the maximum value of 82.14% and 85.13% within 8 h after death at room temperature in Taki, *Channa punctatus* and Shol, *Channa striatus*, respectively, while in ice stored condition values reached the maximum of 89.09% within 9 h in Taki and 89.52% within 10 h in Shol [20]. Rigor mortis in Thai-Pangas, on the other hand, started an hour after death in both the iced and room temperature conditions, and the rigor index value reached 72.23% after 8 h and 85.5% after 5 h, respectively [7]. The preharvest handling of the fish, the stress undergone before slaughter, and the biological state of the fish are additional factors that influence the progression of rigor mortis. The physiological state of the muscle before harvest and slaughter will have an impact on the postmortem muscle biochemistry of the fish [21]. The biochemical process that causes rigor mortis is the same in the muscles of many different fish species and warm-blooded animals, although the onset and rate of progression vary from species to species, and the temperature of the storage medium plays a significant role [22, 23]. It has been demonstrated that lower temperatures can extend the prerigor stage, which in turn can slow the development of rigor mortis and delay the start of it. According to the current study, common carp kept at chilled temperatures showed a prolonged period before developing rigor mortis compared to those kept at ambient temperature. Often, bacterial activity starts around the end of the postmortem stage of death. Owing to this reason, any delay at the beginning of the rigor would enhance the shelf life of the fish [24].

The pH value in fish is considered one of the spoilage indicators [25]. Such an increase in pH can indicate bacterial growth and degradation in quality [16]. Postmortem acidification is one of the natural biochemical processes that take place after an animal dies (the accumulation of lactic acid due to anaerobic glycolysis of glycogen). As a result, postrigor had a pH that was substantially lower than prerigor. [2] noted that postmortem initial pH often differs between species and may show minor changes within the same species.

The results of the present study showed that pH decreased to 6.0 during the initial stages of rigor mortis. The conversion of glycogen to lactic acid, which is the product of anaerobic glycolysis in most fish, is the most viable reason for this, along with increased stress, depleted energy stores (glycogen and ATP), a rapid acceleration of rigor mortis [26, 27]. Fish have a pH value near 7.0 when they are alive, but any glycogen that remains after death is subjected to glycolysis, where it is converted to pyruvic acid and then lactic acid, which makes the flesh of the fish acidic. During the later postmortem changes, pH slightly increased. The pH increase may be attributed to the production of volatile bases, such as ammonia and trimethylamine, as a consequence of bacterial enzymatic activity or the action of autoenzymes [28, 29]. The results of the current study showed that, for fish held at ambient and chilled temperatures, the pH gradually increased after 30 h because of the formation of basic compounds, eventually reaching 7.2 and 7.0 after 40 h, respectively.

The current study found that rigor mortis gradually decreased the levels of water and protein in common carp fish, although this effect was nonsignificant (*p* > 0.05) and increased the levels of fat and ash. The content of fish proteins and fats reflects their nutritional values. [4] pointed out that the period onset of rigor mortis in Nile tilapia and catfish is less in fish preserved at laboratory temperature compared to fish kept in ice and showed that the process of rigor mortis is affected by several different factors including the chemical composition and season of the year, where they recorded a decrease in protein value during prolonged fish storage. The decrease in crude protein during rigor mortis could be due to the slow denaturation of the crude protein to more volatile compounds such as total volatile bases (TVBs), trimethyl amine (TMA), hydrogen sulfide, and ammonia. These findings were in line with the results reported by Jafari [30]. The decrease in protein in fish could be due to reduction because of autolytic degradation combined with endogenous enzymes and bacteria [31]. Fish lipids deteriorate on postmortem by two distinct reactions—hydrolysis and oxidation. Ahmad et al. [32] reported that the various reactions are either nonenzymatic (oxidative) or catalyzed by microbial enzymes or by intracellular or digestive enzymes from the fish itself (hydrolytic changes). The findings can be applied to define quality criteria for this species, and they should help fish farmers and processors create more successful processing and marketing strategies. The present study illustrates the postmortem changes in the muscles of common carp fish throughout postcatch processing.

## 5. Conclusion

The present study highlights the postmortem changes in the muscle of common carp (*Cyprinus carpio*) during processing, specifically focusing on the development of rigor mortis. The rigor mortis index was calculated using a specific formula based on physical measurements before and after its onset. pH changes and fish muscle composition were also analyzed under controlled conditions. The results highlight the significance of these factors in determining fish quality, offering insights to improve aquaculture practices and processing methods. The findings establish quality standards essential for fish farmers and marketers, promoting better product quality and sustainability.

## Figures and Tables

**Table 1:** The chemical composition of common carp during rigor mortis at chilled temperature (0°C) and ambient temperature (25°C).

**Chemical composition**	**Before rigor mortis**	**After rigor mortis**	
		**Chilled temperature (0°C)**	**Ambient temperature (25°C)**
Moisture (%)	76.98 ± 1.04^a^	76.82 ± 0.97^a^	76.71 ± 1.23^a^
Protein (%)	15.92 ± 0.84^a^	15.23 ± 0.58^a^	15.41 ± 0.53^a^
Fat (%)	5.42 ± 0.27^a^	5.87 ± 0.23^a^	5.94 ± 0.31^a^
Ash (%)	1.86 ± 0.03^a^	1.98 ± 0.07^a^	1.93 ± 0.06^a^

*Note:* Similar letters in rows mean there are no significant differences (*p* > 0.05).

## Data Availability

The data that support the findings of this study are available from the corresponding author upon reasonable request.
